# Muscular Rheumatism

**Published:** 1868-10

**Authors:** 


### MUSCULAR RHEUMATISM.

I have noticed in the Journals that the treatment of cases denominated Muscular Rheumatism, including lumbago, pleurodinia, sciatica, etc., is a matter not well settled. In a late number of the *Lancet*, the course pursued in three of the London Hospitals was given, differing from each other, and not embracing the course that I have found successful in quite a number of cases this year, my own included.

Many years ago, Mr. Carmichael, the celebrated surgeon of Dublin, had sciatica gradually creep on him till he was entirely disabled. For some little time, if I mistake not, before he yielded to it, he had to be carried from his carriage to his lecture-room. He had been in the habit of working very hard in his profession, eight hours a day, from breakfast to dinner, without any form of lunch. This proved too much for him, as it probably will for any man.

When he concluded to be treated efficiently, he came to a hot spring in the Pyrenees, and had a warm bath every day, with a dose of Pil. Hydrarg. every night, and Seidlitz powder in the morning. This course restored him the use of his limb within a reasonable time. I have tried a similar course with the best effect in old, confirmed cases.

But the remedies I use in acute cases are calomel in small doses, 1 or 2 grains, with gum Guaiac, and Dover's powder, three times a day. The Dover's powder in doses sufficient to soothe the pain, 3 to 5 grs., and the Guaiac in 8 or 10 gr. doses. These I accompany with a warm water (with salt) sponge-bath every morning, to be followed by good rubbing, with dry towels. A cathartic of castor oil once in two or three days, if the powders do not act sufficiently, and sometimes if they do act some, is very useful.

With this course my cases have uniformly yielded. It is quite possible that belladonna would prove a better remedy in these cases.

In my case, the pain commenced at the sacro-iliac junction, and extended across the back, affecting the lumbar muscles. It was attended with a general failure of the secretions, gradual whitening of the tongue, and increased heat of the skin. The remedies gave me relief in a very few days. F. B. E.

HASTINGS, MIN., *Sept.* 14, 1868.

---

An autopsy has been performed at Bellevue Hospital on a body that had been perfectly preserved for 72 days by means of carbolic acid; still another public autopsy took place upon the body of a patient who had died 107 days previously, and had been preserved in a similar manner, with the same highly satisfactory results.

